# Development and Evaluation of a Rapid Antigen Detection and Serotyping Lateral Flow Antigen Detection System for Foot-and-Mouth Disease Virus

**DOI:** 10.1371/journal.pone.0134931

**Published:** 2015-08-13

**Authors:** Kazuki Morioka, Katsuhiko Fukai, Kazuo Yoshida, Rie Kitano, Reiko Yamazoe, Manabu Yamada, Tatsuya Nishi, Toru Kanno

**Affiliations:** Exotic Disease Research Station, National Institute of Animal Health, National Agriculture and Food Research Organization, Tokyo, Japan; University of Rochester, UNITED STATES

## Abstract

We developed a lateral flow strip using monoclonal antibodies (MAbs) which allows for rapid antigen detection and serotyping of foot-and-mouth disease virus (FMDV). This FMDV serotyping strip was able to detect all 7 serotypes and distinguish serotypes O, A, C and Asia1. Its sensitivities ranged from 10^3^ to 10^4^ of a 50% tissue culture infectious dose of each FMDV stain; this is equal to those of the commercial product Svanodip (Boehringer Ingelheim Svanova, Uppsala, Sweden), which can detect all seven serotypes of FMDV, but does not distinguish them. Our evaluation of the FMDV serotyping strip using a total of 118 clinical samples (vesicular fluids, vesicular epithelial emulsions and oral and/or nasal swabs) showed highly sensitive antigen detection and accuracy in serotyping in accordance with ELISA or RT-PCR. To the best of our knowledge, this is the first report on any FMDV serotyping strip that provides both rapid antigen detection and serotyping of FMDV at the same time on one strip without extra devices. This method will be useful in both FMD-free countries and FMD-infected countries, especially where laboratory diagnosis cannot be carried out.

## Introduction

Foot-and-mouth disease (FMD) is one of the most highly contagious viral diseases, and causes devastating economic damage in the countries affected by it. FMD is caused by foot-and-mouth disease virus (FMDV), which belongs to the *Aphthovirus* genus of the family *Picornaviridae*, and consists of 7 immunologically distinct serotypes: O, A, C, Asia1, and South African Territories (SAT) 1, 2 and 3. Within a single serotype, there may be many different topotypes [1]; serotype A in particular displays a large number of topotypes [2]. In Asia, type O, A and Asia1 viruses have prevalently existed and the disease is endemic or sporadic in main countries [3]. Additionally, there have been some cases of FMD whose serotype is unknown in South East Asia [3]. Although outbreaks of the SAT serotypes have been largely limited to the African continent, it has been reported that the SAT2 serotype invaded Middle Eastern countries in 2000 and 2012 [4].

In an FMD outbreak, antigen serotyping should be conducted immediately in order to implement the appropriate plan for disease control (e.g., preparing vaccine and immunological diagnosis kits for the serotype in question). To serotype FMDV, indirect sandwich enzyme-linked immunosorbent assay (ELISA), which is mentioned in the World Organization for Animal Health (OIE) *Terrestrial Manual 2012* [5], may be used, however it lacks sufficient sensitivity and specificity [6–9]. Although some studies have reported serotyping by reverse transcription-polymerase chain reaction (RT-PCR) or real-time RT-PCR in some regional endemic FMD strains, the mutability of viral RNA makes it difficult to apply either of these as a universal method [10]. Moreover, the recent activation of world trade and human transportation might change the epidemic styles of trans-boundary emerging diseases. The possibility of new outbreaks of the different topotypes in places where they have never previously occurred is increasing. We have developed a monoclonal antibody (MAb)-based direct sandwich ELISA (MSD-ELISA), which demonstrates higher sensitivity than those of current indirect sandwich ELISA methods reported in previous studies [8,9]. ELISA is a useful tool for antigen detection, however this should be carried out in a laboratory with the appropriate apparatus.

In this report, we developed a lateral flow assay using MAbs (FMDV serotyping strip), which allows for rapid FMD antigen detection for all 7 serotypes and FMD serotyping for types O, A, C and Asia1, specifically in the field, and especially in countries where laboratory diagnosis cannot be carried out, in vast countries in which it would take a long time to transport clinical samples to a laboratory, and/or in countries lacking transportation facilities.

## Materials and Methods

### Cells and viruses

The virus strains FMDV O/JPN/2000 (ME-SA topotype, Pan-Asia lineage) [11–13], O/JPN/2010 (SEA topotype, Mya-98 lineage) [14], O1 Manisa (TUR 8/69) (ME-SA topotype, Pan-Asia lineage), O1 BFS 1860 (Euro-SA topotype), O/TAW/97 (Cathay topotype) [15, 16], O/TUR/5/2009 (ME-SA topotype, Pan-Asia2 lineage), A15 TAI 1/60 (Asia topotype), A22 IRQ 24/64 (Asia topotype), A/IRN/1/2011 (Asia topotype, Iran-05 ^FAR-11^ lineage), A/TAI/10/2011 (Asia topotype, Sea-97 lineage), C PHI 7/84 (Euro-SA topotype), Asia1 Shamir (ISR 3/89) (Asia topotype), Asia1/TUR/49/2011 (Asia topotype, Shindh-08 lineage) [17, 18], SAT1/KEN/117/2009 (topotype I (NWZ)), SAT2/SAU/6/2000 (topotype VII), SAT3/ZIM/3/83 (topotype I (SEZ)) and swine vesicular disease virus (SVDV) J1/73 [19, 20] were grown on monolayers of IBRS-2 [21] and/or BHK-21 [22] cells and used for this study.

### Monoclonal antibodies

In the present study, MAbs producing hybridomas 13F1 and 2A1 were established against C PHI 7/84 and A/IRN/1/2011, respectively, by the general method as previously described [12]. MAb 1H5 and MAb 70C4 originating from O/JPN/2000, MAb16C6 originating from A15 TAI 1/60, and MAb 12C7 originating from Asia1 Shamir (ISR 3/89) were used in this study [8, 9].

### Conjugation of MAb 1H5 with colloidal gold

A mixture of 0.1 ml of purified MAb 1H5, which reacts with all 7 serotypes of FMDV (200 μg/ml) and 0.8 ml of colloidal gold (pH 8.0) (Winered Chemical Corporation, Tokyo, Japan) was stabilized for 8 min at room temperature. Next, 0.1 ml of 10% bovine serum albumin (BSA) and 0.01 ml of 5% polyethylene glycol (PEG) were added, and the mixture was centrifuged at 10,000 rpm at 20°C for 30 min. After removal of the supernatant, the colloidal gold-labeled MAb 1H5 was resuspended with 1.5 ml of phosphate buffered saline (PBS) containing 0.5% BSA and 0.05% PEG, centrifuged again under the same conditions mentioned above, and resuspended with 20 mM Tris-HCl (pH 7.5) containing 10% trehalose, 10% BSA and 5% PEG. After the density was adjusted to 2.0 of OD_520_, 50 μl of the colloidal gold labeled MAb 1H5 (G-MAb) was freeze-dried in a 2-ml tube.

### Production of the FMDV serotyping strip

Anti-FMDV MAbs against each serotype, MAb 70C4 (type O), 16C6 and 2A1 (type A), 13F1 (type C) and 12C7 (type Asia1), multi-serotype reactive MAb 1H5 and AffiniPure goat anti-mouse IgG (H+L) (Jackson ImmunoResearch Laboratories, Inc., West Grove, PA, USA) were suspended with 5 mM phosphate buffer (pH 7.5) in 1000–1500 μg/ml, and applied on a nitrocellulose membrane (Hi-Flow Plus HF135; Millipore, Billerica, MA, USA) using immunoliner 200 (System Biotics, Kanagawa, Japan) at 0.8 μl/cm. The antibodies on nitrocellulose membranes were dried at 50°C for 30 min, dipped in 0.05 M boric acid with 0.5% casein (pH 8.5) for 30 min at room temperature, dipped in 50 mM Tris-HCl (pH 7.4) with 0.5% sucrose and 0.05% sodium cholate for 30 min at room temperature, and dried at room temperature overnight.

The cellulose fiber membrane (Cellulose Absorbent Sample Pads; Millipore), which was cut to a width of 1 cm, dipped in 100 mM Tris-HCl (pH 8.0) with 1.6 mM ethylenediaminetetraacetic acid (EDTA) for blocking against non-specific reactions due to contamination of saliva or hemolytic fluid and dried overnight at room temperature, was used as an absorption pad, and stuck with adhesive tape on the downstream side of the nitrocellulose membrane, overlapping by 5 mm (ARcare 7815; Adhesives Research, Glen Rock, PA, USA). A cotton membrane (GE Healthcare Life Sciences, Little Chalfont, UK) 2 cm in width was used as an absorption pad and stuck with adhesive tape on the upstream side of the nitrocellulose membrane, overlapping by 5 mm. Constructed membranes were then cut at a width of 0.5 cm, and the strips were stored at room temperature under low humidity.

### Monoclonal antibody-based sandwich direct ELISA

MSD-ELISA was established and described in our previous reports [8, 9], and the protocol is described in detail [8]. In the present study, OD values over 0.1 were deemed positive.

### Indirect sandwich ELISA

Indirect sandwich (IS) ELISA [23], which is a method described in the OIE manual [5], was carried out according to the relevant instruction manuals. For the purposes of the present study, OD values over 0.1 were considered positive.

### Statistics

The Pearson’s chi-square test was used to analyze the statistical significance of the differences in virus detection rates between IS-ELISA and the FMDV serotyping strip. To analyze correlations between the OD values of MSD-ELISA and the mABS values of the FMDV serotyping strip, Pearson’s correlation coefficient was used.

### RT-PCR

For the RT-PCR for the detection of FMDV nucleic acid, the SuperScript III One-Step RT-PCR System with Platinum Taq DNA Polymerase (Invitrogen, Waltham, MA, USA) and primers for the 3D region were used [15].

### Lateral flow antigen detection commercial kit

A Svanodip FMDV-Ag kit (Boehringer Ingelheim Svanova, Uppsala, Sweden) was used following the manufacturer’s instructions. A total of 170 μl of samples diluted with sample buffer were added to the device and, after 10 min, mABS was measured using the Immunochromato-Reader C10066.

### Clinical samples

A total of 16 samples of vesicular fluids collected from pigs inoculated with FMDV O/JPN/2000 and A15 TAI 1/60 were used for each antigen detection method. A total of 53 samples of vesicular epithelial tissues collected from pigs and cattle inoculated with FMDV O/JPN/2010 were used for each antigen detection method.

In addition to the experimental samples, a total of 49 RT-PCR-positive samples (41 oral and/or nasal swabs soaked in approximately 10-times volumes of PBS (about 2 ml) and 12 samples of 10% emulsion of homogenized epithelial tissues) collected from cattle and pigs involved in the 2010 type O FMD outbreak in Japan caused by O/JPN/2010 (SEA topotype) [14] were used for comparative studies.

Field samples (vesicular epithelium and vesicular lesion’s swab) were submitted from Miyazaki Prefecture, Japan, for diagnosis of the 2010 FMD in Japan.

### Ethics statement

The animal experiments carried out in the present study were approved by the Ethics Committee of the National Institute of Animal Health, Japan (approvals #09–029, 14–060, and 14–080). The field samples used in this study were submitted from Miyazaki Prefecture, Japan, for diagnosis of the 2010 FMD in Japan. These vesicular epithelial tissues were collected by veterinarians in accordance with the guidelines specified in the Act on Domestic Animal Infectious Diseases Control.

### The FMD serotyping strip

One hundred μL of samples diluted with PBS were added to the tube containing the freeze-dried G-MAb, and the FMDV serotyping strip was then dipped into the tube. After 12 min, the lines were interpreted by visual observation and/or the milli absorbance (mABS) of the lines was measured using an Immunochromato-Reader C10066 (Hamamatsu Photonics, Shizuoka, Japan). With respect to visual interpretation, mABS “from 10 to 15” and “over 15” were deemed equivalent to “weak positive” and “positive,” respectively.

#### Antigen detection for all seven serotypes and serotyping (O, A, C and Asia1)

A total of 17 viruses, 16 strains of FMDV and the SVDV J1 strain, were adjusted to almost 10^6^ of 50% tissue culture infectious dose (TCID_50_)/100 μL with PBS, and those of 100 μL were applied for the FMD serotyping strips.

#### Detection limits of the FMD serotyping strip

Ten-hold serial dilutions were prepared with PBS or running buffer for Svanodip against the 16 FMDV strains as follows: FMDV O/JPN/2000, O/JPN/2010, O1 BFS 1860, O1 Manisa (TUR 8/69), O/TUR/5/2009, O/TAW/97, A15 TAI 1/60, A22 IRQ 24/64, A/IRN/01/2011, A/TAI/10/2011, C PHI 7/84, Asia1 Shamir (ISR 3/89), Asia1/TUR/49/2011, SAT1/KEN/117/2009, SAT2/SAU/6/2000 and SAT3/ZIM/3/83, and applied to the FMD serotyping strip (100 μl) and Svanodip (170 μl). The total of applied virus antigens of Svanodip were 1.7 times as much as that of the FMD serotyping strip. Only those for type SAT3 were applied from 10^6^ TCID_50_.

#### Evaluation of the FMD serotyping strip using clinical samples

The FMD serotyping strips were evaluated using 10 μl of vesicular fluid from pigs inoculated with O/JPN/2000 or A15/TAI/1/60, or almost 10% (1 to 10%) vesicular epithelial emulsions of pigs and cattle inoculated with O/JPN/2010 and of animals infected in the 2010 outbreak in Japan caused by O/JPN/2010, and the results were compared with those obtained using other antigen detection methods, specifically MSD-ELISA, IS-ELISA and RT-PCR. IS-ELISA was not performed for vesicular fluids because of insufficient sample volume. In the present study, mABS values over 15 were judged positive samples, and this almost correlated with visual inspections.

In this evaluation, the positions of the serotype-specific antibodies were rearranged from O, A, Asia1, C (from upstream to downstream) to C, O, A, Asia1 to inspect non-specific reactions due to contamination of hemolytic fluid or other contamination.

## Results

### Antigen detection for all seven serotypes and serotyping (O, A, C and Asia1)

**Fig 1:**
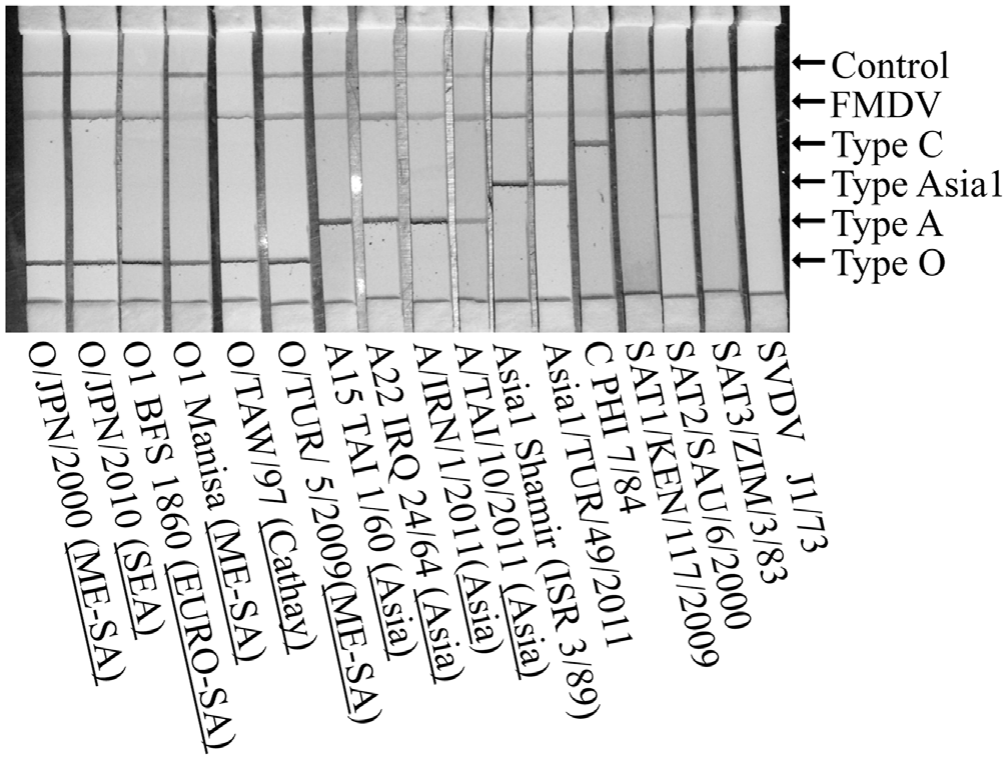
FMDV serotyping on one strip. The result of reactivity of the FMD serotyping strip against seven serotypes of FMDVs and SVDV. One hundred μL of a total of 17 viruses of almost 10^6^ TCID_50_ were added to the tube containing the freeze-dried G-MAb, and the FMDV serotyping strip was then dipped into the tube. After 12 min, the lines were interpreted.

The FMDV serotyping strips were able to detect all 16 strains of the 7 serotypes of FMDVs including recent epidemic strains, and were able to distinguish serotypes O, A, C and Asia1, but did not react to SVDV. However, when a high titer of SAT2 was applied, a weak non-specific line was observed on the serotype A line (Fig 1).

### Sensitivities of the strip

**Fig 2:**
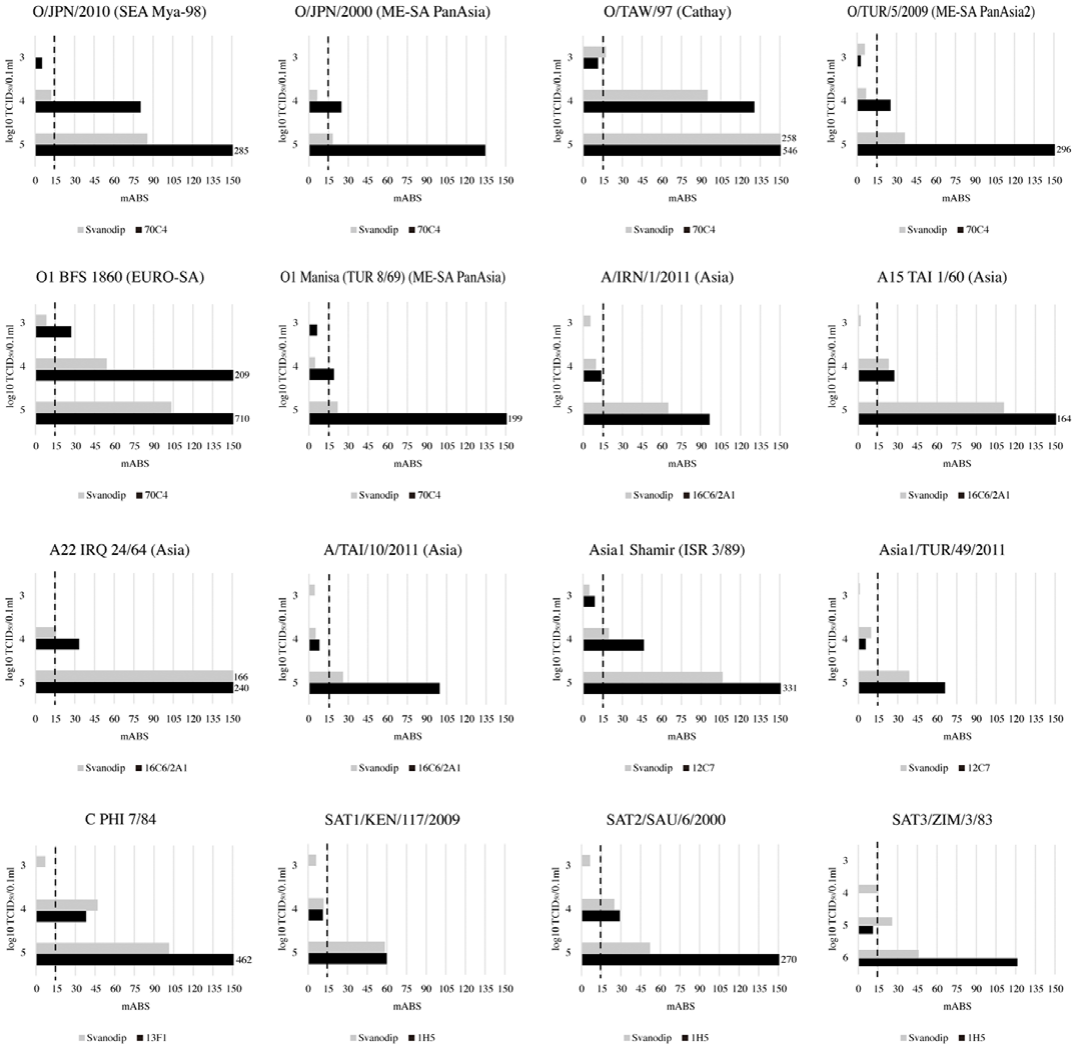
The sensitivities of the FMD serotyping strips. The sensitivities of the FMD serotyping strip and Svanodip against each FMDV strain were compared. Only type SAT3 was applied from 10^6^ TCID_50_. Closed bars show the mABS of each serotype-specific line of the FMD serotyping strip. Shaded bars show the mABS of Svanodip. The dashed lines on the graphs show 15 mABS lines judged positive by visual inspection. The numbers on the right edge of the graph show mABS over 150.

The antigen detection limits of each serotyping line of the FMDV serotyping strip were found to be almost equal to 10^3^ to 10^4^ TCID_50_ of each virus stain (Fig 2). For O/JPN/2010 (SEA), the FMDV serotyping strip showed a strong positive line at 10^4^ TCID_50_ and a doubtful line at 10^3^ TCID_50_, while the Svanodip showed a very weak positive at 10^4^ TCID_50_. The detection limits of both the FMDV serotyping strip and Svanodip for O/TAW/97 and O1 BFS1860 were almost 10^3^ TCID_50_. The detection limits for the ME-SA topotype (O/JPN/2000, O1 Manisa, O/TUR/5/2009) of the FMDV serotyping strip and Svanodip were 10^4^ and 10^5^ TCID_50_, respectively. In the case of serotype A strains, the detection limits of the FMDV serotyping strip and Svanodip were around 10^4^ TCID_50_. The detection limits of the FMDV serotyping strip and Svanodip for Asia1 Shamir (ISR 3/84) and Asia1/TUR/2011 were 10^4^ and 10^5^ TCID_50_, respectively. The detection limits of both the FMDV serotyping strip and Svanodip for C/PHI/7/84, SAT1/KEN/2009 and SAT2/SAU/2000 were 10^4^ TCID_50_. The FMDV serotyping strip showed high sensitivity to the serotype O ME-SA topotype compared to Svanodip. On the other hand, the FMDV serotyping strip was not sensitive to SAT3, at a detection limit of 10^5^ TCID_50_, while Svanodip were able to detect 10^4^ TCID_50_.

### Evaluation of the FMD serotyping strip using clinical samples

As shown in Table 1, the FMDV serotyping strip was able to detect FMD antigens on the target serotype lines against all 16 samples of vesicular fluid (9 samples for A15 TAI 1/60 and 7 samples for O/JPN/2000), 90.2% (55/61) samples of 10% vesicular epithelial emulsions collected from experimental infection of O/JPN/2010, and 68.3% (28/41) of oral and or nasal swabs from the 2010 outbreak in Japan caused by O/JPN/2010. The FMDV serotyping strip was found to be more sensitive than IS-ELISA against vesicular epithelial emulsions and oral and/or nasal swabs (p<0.01). The correlation coefficient between mABS and the OD of MSD-ELISA was significantly high (R = 0.67).

**Table 1:** Comparison of the results of antigen detection using clinical samples.

virus strain	Experimental or field	animals	samples	FMDV Serotyping Strip (O or A)	MSD-ELISA^a^ (O or A)	IS-ELISA^b^ (O)
A15 TAI 1/60	experimental	pig	VF^c^ ^,^ ^d^	100% (9/9)^e^	100% (9/9)^e^	Not tested
O/JPN/2000	experimental	pig	VF	100% (7/7)	100% (7/7)	Not tested
O/JPN/2010	experimental	pig	VEE^f^	100% (37/37)	100% (37/37)	67.6% (25/37)^e^
O/JPN/2010	field	pig	VEE	100% (4/4)	100% (4/4)	100% (4/4)
O/JPN/2010	field	pig	SW^g^	55.6% (5/9)	100% (9/9)	0.0% (0/9)
		subtotal		93.9% (62/66)	100.0% (66/66)	58.0% (29/50)
O/JPN/2010	experimental	cattle	VEE	68.8% (11/16)	87.5% (14/16)	25.0% (4/16)
O/JPN/2010	field	cattle	VEE	75.0% (3/4)	75.0% (3/4)	0.0% (0/4)
O/JPN/2010	field	cattle	SW	71.9% (23/32)	100% (32/32)	9.4% (3/32)
		subtotal		71.2% (37/52)	94.2% (49/52)	13.5% (7/52)
		total		83.9% (99/118)	97.5% (115/118)	35.3% (36/102)

^a^ MSD-ELISA is MAb-based antigen detection ELISA

^b^ IS-ELISA is indirect sandwich ELISA supplied by the World Reference Laboratory

^c^ Vesicular fluid

^d^10 μl of vesicular fluid were added to 90 μl of running buffer

^e^The number of positive samples by each antigen detection method/the number of positive samples by RT-PCR

^f^ Vesicular epithelial emulsion

^g^ Oral and/or nasal vesicular lesion swabs.

## Discussion

In the present study, we developed an FMD serotyping strip that enables rapid antigen detection for all 7 serotypes and serotyping for O, A, C and Asia1, including recent epidemic FMDVs. This FMD serotyping strip allows for rapid antigen detection and 4 types of serotyping of FMDV without extra devices.

Although antigen detection ELISA requires that samples of each serotype are added to detecting wells, this FMD serotyping strip is able to perform simultaneous detection and serotyping of FMDV on a single strip. Furthermore, our lateral flow system detects not only FMDV but also type-specific FMDV. This not only increases sensitivity but also counteracts the shortcomings of MAb, which recognizes a single epitope that depends on broad intra-type reactivity.

In Southeast Asia, there have been many cases in which the causative FMDV serotype was unknown [3]. The present method will be useful not only in FMD-free countries but also in FMD-infected countries, especially where laboratory diagnosis is not possible. Thus, the FMD serotyping strip will prevent delay of serotyping reports to the OIE and we will be able to share more precise information about FMD epidemics.

The advantage of using MAbs is that it is possible to select highly efficient MAbs for high specificity and sensitivity. However, a MAb recognizes a single epitope, and thus the broad intra- and intertype reactivity of the MAbs should be evaluated. To confirm the relativities of FMDV epidemic strains and our MAbs, we introduced the recent epidemic FMDV serotype O, A and Asia1 strains O/TUR/5/2009, A/IRN/01/2011, A/TAI/10/2011, Asia1/TUR/49/2011, and serotypes SAT1, SAT2 and SAT3 as follows: SAT1/KEN/117/2009, SAT2/SAU/6/2000 and SAT3/ZIM/3/83. MAbs 70C4 and 12C7 were able to react to the recent epidemic FMDV strains of serotypes O and Asia1, respectively. MAb 1H5 was able to react to the recent epidemic FMDV strains including serotype SAT1, 2 and 3. Therefore, the multi-serotype detection line is able to be warranty for SAT1, 2 and 3, and in view of mismatch of each serotype specific MAbs. In our previous study, we used MAb 16C6 as a detecting antibody for MSD-ELISA for type A FMDV, however it did not react to A/IRN/01/2011, although it react to A/TAI/10/2011. The strain A/IRN/01/2011 belongs to the A-Iran-05 lineage, which appeared in 2005 as a new lineage of FMDV serotype A [24, 25]. That strains spread throughout Iran and moved westward into Saudi Arabia and Turkey, and were also reported in 2006 in Pakistan, and in 2007 in Jordan [25]. To solve this problem, we produced MAb 2A1 by A/IRN/01/2011 as the antigen, and this MAb was able to react to A/IRN/01/2011 as well as to A22 IRQ 24/64. Therefore, in the present study, we used both MAb 16C6 and 2A1 as cocktails of the antigen detection MAb on the membrane.

We used oral swabs for samples for MSD-ELISA in our previous study [9], however, we found that some oral and/or nasal vesicular lesion swabs from 2010 inhibited chromatography flow in the present study (data not shown). Saliva is expected to be a useful sample for detecting antigens, especially from preclinical infected animals [26] and subclinical infected animals such as those that are vaccinated-infected or have low pathogenic FMDV. For instance, the cattle in the 2000 FMD outbreak in Japan did not show any distinct vesicles on the feet, or in or around the oral and nasal cavities. [11–13]. In addition, even in typical clinical cases such as those in 2010, almost all samples were oral and/or nasal vesicular lesion swabs from the outbreak area because the vesicles readily ruptured and it was difficult to collect epithelial tissues.

On the other hand, using vesicular fluids and/or epithelial emulsions as a sample has the advantage that the sample would include a high titer of virus, and antigens can be detected a long time after the appearance of lesions. The present FMDV serotyping strip was able to detect antigens from 4 to 13 days (the end of the experiment) post-inoculation when the lesions were almost healed (Table 1; sampling date not shown).

In this study, the correlation coefficient between mABS of the FMDV serotyping strip and the OD of MSD-ELISA was significantly high (R = 0.67) and this strip was found to be more sensitive than IS-ELISA (p<0.01). As described in our previous study [9], the difference of sensitivity was probably due to the ability of MAb that we used in this system, much more than those of rabbit and guinea pig anti-sera in IS-ELISA. In addition, concerning specificity, the FMD serotyping strip showed negative results against a total of 21 of RT-PCR negative samples (14 of oral swabs or saliva and 7 of epithelial emulsions) (data not shown).

Further study is ongoing, and we are trying to establish serotype-specific MAbs against SAT1, 2 and 3, and apply them in the lateral flow assay for the detection and serotyping of all 7 serotypes of FMDVs.
